# A Modified Method for Determination of Lumefantrine in Human Plasma by HPLC-UV and Combination of Protein Precipitation and Solid-Phase Extraction: Application to a Pharmacokinetic Study

**DOI:** 10.4137/aci.s4431

**Published:** 2010-03-29

**Authors:** Liusheng Huang, Patricia S. Lizak, Anura L. Jayewardene, Florence Marzan, Ming-Na Tina Lee, Francesca T. Aweeka

**Affiliations:** Department of Clinical Pharmacy, School of Pharmacy, University of California, San Francisco, CA 94110, USA.

**Keywords:** HPLC-UV, lumefantrine, halofantrine, protein precipitation, SPE

## Abstract

An HPLC-UV method was developed and validated for the determination of lumefantrine in human plasma. Lumefantrine and its internal standard halofantrine were extracted from plasma samples using protein precipitation with acetonitrile (0.2% perchloric acid) followed by solid-phase extraction with Hypersep C_8_ cartridges. Chromatographic separation was performed on a Zorbax SB-CN HPLC column (3.0 × 150 mm, 3.5 μm) with water/methanol (0.1% TFA) as the mobile phases in a gradient elution mode. Detection was performed using UV/vis detector at λ = 335 nm. The method showed to be linear over a range of 50–10,000 ng/mL with acceptable intra- and inter-day precision and accuracy. The mean recoveries were 88.2% for lumefatrine and 84.5% for the I.S. The internal standard halofantrine is readily available from commercial sources. This method was successfully applied to a pharmacokinetic interaction study between a first-line antimalarial combination (artemether—lumefantrine) and antiretroviral therapy.

## Introduction

Malaria is a serious and sometimes fatal protozoan disease caused by malaria parasites (*Plasmodium vivax, ovale, falciparum and malariae*) and transmitted by the female *Anopheles* mosquitoes. Symptoms of malaria include fever, chills, sweats, headache, nausea, and vomiting. Each year, malaria afflicts an estimated 350–500 million people and leads to 1.5–2.7 million deaths worldwide. Nine percent of all deaths in children less than 5 years of age are caused by the disease, and this percentage is as high as 20% in sub-Saharan Africa.^1^

Lumefantrine (LF), also named benflumetol and chemically (9z)-2,7-dichloro-9-[(4-chlorophenyl) methylene]-a-[(dibutylamino)methyl]-9H-fluorene-4-methanol, is an aryl alcohol antimalarial first synthesized in the 1970’s by the Academy of Military Medical Sciences, Beijing, China and registered in China for the treatment of malaria in 1987.^2^ The compound is a yellow powder that is poorly soluble in water, oils, and most organic solvents, but soluble in unsaturated fatty acids and acidified organic solvents. LF is extensively bound (>99%) to plasma proteins, mainly high density lipoproteins.^3^ LF as a drug is commercially available only in a fixed-dose combination with artemether (Coartem^®^ or Riamet^®^).^4^ This combination is well tolerated and highly effective and now becoming the most recommended first-line treatment for uncomplicated *falciparum* malaria in African countries.

To support pharmacokinetic interaction study between artemether/LF and an antiretroviral treatment for patients co-infected with malaria and HIV, here we describe an HPLC-UV method to determine LF in human plasma. Previously, Mansor et al reported an HPLC-UV method with a narrow calibration range (25–800 ng/mL) and requiring a large sample volume (1 mL).^5^ Lindegardh and coworkers developed HPLC-UV methods to determine LF in plasma and sampling filter paper.^4^,^6^ However, the recovery of LF was relatively low (60%–75%), and the internal standards used in these methods are not commercially available. Our method is based on a previously published method with the following modifications^6^: (1) Protein precipitation utilized acetonitrile containing 0.2% perchloric acid in place of 1% acetic acid, affording an improved recovery (∼90%). (2) Use of the gradient mobile phase water (0.1% TFA) and methanol (0.1% TFA) which are salt-free solvents, enabled column wash with a high percentage of organic solvent. (3) The internal standard halofantrine is commercially available.

## Experimental

### Reagents and materials

**Figure 1.**
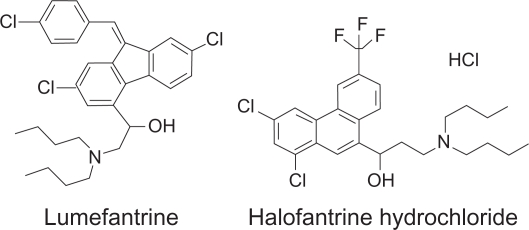
Chemical structures of lumefantrine and halofantrine (the I.S.).

Lumefantrine (Figure 1) and halofantrine (the internal standard, I.S.) were purchased from A.K. Scientific Inc. (Mountain View, CA, USA). Acetonitrile (MeCN), methanol (MeOH), water (H_2_O), Perchloric acid (HClO_4_), and formic acid (HCO_2_H) were obtained from Fisher Scientific (Fair Lawn, NJ, USA). Trifluoroacetic acid (TFA) was purchased from Sigma-Aldrich (St. Louis, MO, USA). All chemicals were of HPLC grade. Water was distilled water, if not mentioned specifically. Human plasma was purchased from Biological Specialty Co. (Colmar, PA, USA).

### Instrumental and analytical conditions

The HPLC system consisted of Waters 1525 binary HPLC pumps, Waters 717 plus autosampler, and Waters 2487 dual λ absorbance detector controlled by Waters Breeze software (Version 3.30 SPA). Separation was achieved on a Zorbax SB-CN HPLC column (150 × 3.0 mm, 3.5 μm, Agilent), equipped with a pre-column filter, used at room temperature. The UV/vis detector was set at 335 nm, and injection volume was 50 μL. The mobile phases were water with 0.1% TFA (A) and MeOH with 0.1% TFA (B) pumped at a flow rate of 0.4 mL/min. The gradient program consisted of linear segments with 68% B (0–4 min), 68%–75% B (4–18 min), 75%–95% B (18–19 min), 95% (19–22 min), 95%–68% B (22–22.5 min), and 68% B (22.5–31 min).

### Preparation of standard and quality control samples

Primary stock solutions of LF and I.S. were each prepared at a concentration of 1 mg/mL in MeCN-water (1:1) containing 0.5% formic acid. These primary solutions were diluted with MeCN-water (1:1) containing 0.1% formic acid to prepare working stock solutions and working solutions. The working solutions of LF were spiked to blank plasma to obtain calibration standards of 50, 100, 250, 500, 1000, 2500, 5000, 7500 and 10000 ng/mL. QC samples were spiked at 120, 900 and 9000 ng/mL by adding the working solutions into blank human plasma. Calibration standards and QC samples were prepared from separately weighted stock solutions. The stock solutions, standards, QC samples, and the I.S. working solution (100 μg/mL) were stored at −70 °C between uses.

### Sample preparation

To a 0.2 mL aliquot of each standard, QC, and blank plasma was added 50 μL I.S. (100 μg/mL halofantrine). The mixture was precipitated with 0.5 mL MeCN containing 0.2% HClO_4_. After vortexing and centrifuging, the sample was poured into a pre-conditioned Hypersep C_8_ solid-phase extraction (SPE) cartridge (50 mg/1 cc, Thermo-Fisher) and pipet-mixed with 0.5 mL pre-loaded water. After pipet-mixing, the sample was drained into the bed and washed with water (1 mL × 3) and subsequently 0.5 mL MeCN-water (2:3) containing 0.1% TFA. The cartridge was then dried under vacuum (∼8 in Hg) for 10 min followed by eluting with 0.5 mL MeOH (0.1% TFA). The eluted sample was dried at 40 °C with a stream of N_2_, reconstituted with 200 μL MeOH- water (68:32) containing 0.1% TFA, and transferred into an autosampler vial.

### Method validation procedure

The method validation was conducted according to the guidelines of NIH funded AIDS Clinical Trials Group (ACTG),^7^ which were developed based on Food and Drug Administration (FDA) guidelines. Calibration curves were obtained by linear regression of the peak area ratio of analyte to internal standard (Y-axis) versus the nominal analyte concentrations (X-axis) with a weighting factor of 1/x. The LLOQ was established using five samples independent of standards to determine accuracy and precision. The signal intensity of the LLOQ was ≥5-fold blank response. Intra-day precision and accuracy were determined by analysis of five replicates of each QC sample (n = 5) at low (120 ng/mL), medium (900 ng/mL), and high (9000 ng/mL) concentration levels extracted with a set of standards in one batch. The same procedure was repeated on five different days with new samples to determine inter-day precision and accuracy (total: n = 25 per concentration level). Precision was reported as relative standard deviation (RSD, %) and accuracy as percent deviation from the nominal concentration (% deviation). Recovery was assessed by comparing the peak area of LF or I.S. from the normally processed plasma samples to the peak area of LF or I.S. from directly injected water-MeCN (1:1) solution with the same concentration of LF or I.S. Specificity of the assay was tested with 6 different sources of human plasma and potential concomitant drugs. The stability of LF in human plasma was evaluated at these conditions: 3 freeze-thaw cycles, storage at −70 °C, room temperature storage (22 °C), and at different steps in the sample preparation process. Each condition was tested with QC samples at low and high concentration levels in triplicates. Fresh samples were used as reference. Stock solutions of LF and I.S. were evaluated at −70 °C and room temperature (22 °C).

### Application to pharmacokinetic study in healthy volunteers

Plasma samples from 13 healthy volunteers were tested with this validated method. The clinical study was conducted at the Clinical and Translational Science Institute Clinical Research Center, San Francisco General Hospital. Each subject received 6 doses of Coartem (80 mg artemether and 480 mg LF) twice daily (study days 1–4), at day 4 blood samples were collected before the sixth dose and at 0.5, 1, 2, 4, 6, 8, 12, 24, 48, 72, 96, 120, 168, 216, and 264 hr post-dose for the analysis of LF. Whole blood samples were drawn into EDTA-containing tubes and centrifuged at 2000 g for 10 min. The resulting plasma samples were stored at −70 °C before analysis.

## Results and Discussion

### Internal standard selection

The ideal I.S. should possess similar behavior with the analyte in the process of sample preparation, LC separation, and detection, and it should not be potentially present in the samples. The I.S. should be eluted near the analyte but not overlap with the analyte unless different detection channels are used.^8^ Efforts on selecting a close analog of LF as the I.S. were limited by commercial availability. Finally halofantrine was selected as the I.S. Halofantrine is readily available commercially and detectable at 335 nm. The recovery of the IS from sample preparation was similar with LF, with a retention time approximately 6 min less than LF. Sporadically interfering peaks were observed, but with a relative intensity less than 5% of I.S. signal at the final I.S. concentration. The I.S. concentration affects the fitting of calibration curve. Too high concentration of I.S. results in large error at lower end of the calibration curve. The I.S. concentration was selected based on its detection signal equivalent to the signal of the analyte at a concentration between the lower one-third and the middle of the calibration range in terms of magnitude. The I.S. concentration in the final injection solution was 25 μg/mL. Based on its UV response at 335 nm, 25 μg/mL I.S. was equivalent to ∼700 ng/mL LF, which was close to the middle point of the calibration curve.

### LC optimization

**Figure 2.**
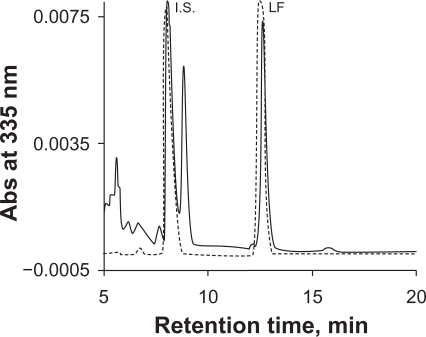
Chromatograms of lumefantrine and the I.S. with MeCN-water (55:45) containing 0.1% TFA as the mobile phase. Dash gray line represented a sample in mobile phase solvents, solid black line represented a sample in plasma. A peak interfering with the I.S. was observed from the plasma sample.

**Figure 3.**
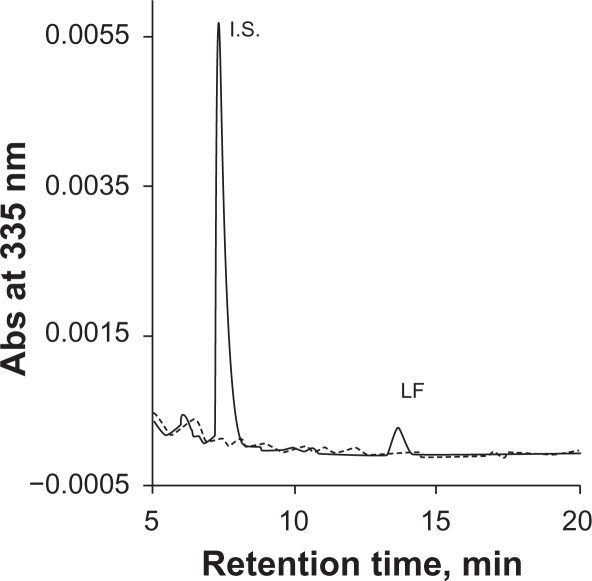
Representative chromatogram of lumefantrine and the I.S. with MeOH-water containing 0.1% TFA as mobile phase in the final method. Dash gray line is for blank plasma, solid black line is for an LLOQ sample in plasma.

Phosphate buffer is commonly used as a mobile phase component in HPLC-UV assay to maintain constant pH. However, phosphate salts tend to precipitate in the solvent line with increasing organic solvent, resulting in instrument failure. We used 0.1% TFA instead of phosphate buffer to maintain an acidic condition in order to minimize peak tailing. Initially MeCN-water (55:45) with 0.1% TFA was used as mobile phase. However, an interfering peak partially co-eluted with the I.S. (Figure 2). This interference was eliminated by switching MeCN to MeOH. A drawback to using MeOH as a mobile phase is higher system pressure compared to MeCN. During the run, the LC system pressure ranged from 2500 to 3500 psi. A representative chromatogram of LF and the I.S. from the final method is showed in Figure 3.

### Sample preparation

It has been reported that over 99% LF is bound to plasma proteins.^3^ Lindegardh and coworkers found protein precipitation prior to SPE increased recovery dramatically compared to SPE alone.^6^ The recovery in the reported method was in the range of 60%–75%. The recovery from liquid-liquid extraction was reportedly over 90%.^9^ Loss of LF may occur at the protein precipitation or SPE step. No improvement of recovery was achieved with larger volumes of the elution solvent. Therefore, LF was expected to be lost during protein precipitation. Precipitation of plasma samples with 1% acetic acid in MeCN yielded large particles of precipitate. This may cause inefficient release of LF from the plasma proteins. Interestingly, addition of a stronger acid (0.2% perchloric acid) in MeCN generated fine particles of the precipitate, which improved the recovery significantly (up to 90%) (Table 1). In addition, a 10-min drying step was added prior to elution of LF from the SPE column as residual water could compromise the elution power of methanol.

### Method validation

Linearity: A calibration curve was calculated and fitted by 1/x weighted regression of the peak-area ratios of LF to I.S. versus the concentrations of LF over the range of 50–10,000 ng/mL. A weighting factor of 1/x is necessary because of the wide concentration range of the calibration curve. The mean back-calculated values of standards for 5 run days were all within the acceptable range (<20% for LLOQ, < 15% for other standards) (Table 2).

Precision and accuracy: The intra-day and inter-day precision (RSD) was all within 15% over the calibration range (Table 3). The intra- and inter-day accuracy (% dev) also met the guidelines that required <20% for LLOQ and <15% for QC samples.^7^

Specificity: We tested six different sources of human plasma and 12 potential concomitant drugs: nevirapine, lopinavir, ritonavir, zidovudine, lamivudine, efavirenz, chloroquine, sulfamethoxazole, trimethoprim, artemether, dihydroartemisinin, tenofovir. No significant interfering peaks were observed during the retention times of LF and I.S. at the wavelength of detection (λ = 335 nm), indicating high specificity and selectivity of the method (see supplemental data Figure S1 and S2).

Stability: LF was stable in plasma at the tested conditions, and LF stock solution was stable for at least 9 months in −70 °C (Table 4). The processed sample in the reconstitution solvent was stable for at least 3 days. However, the processed sample in glass tubes without reconstitution solvent was only stable for 1 day. If the extracted sample was reconstituted after 2 days, recovery dropped to approximately 60%. The lower recovery rate was most likely a result of acid-catalyzed LF degradation due to residual acid (HClO_4_ or TFA) in the sample. It is also possible that the LF-acid salt is unstable.

### Application to pharmacokinetic study in healthy volunteers

**Figure 4.**
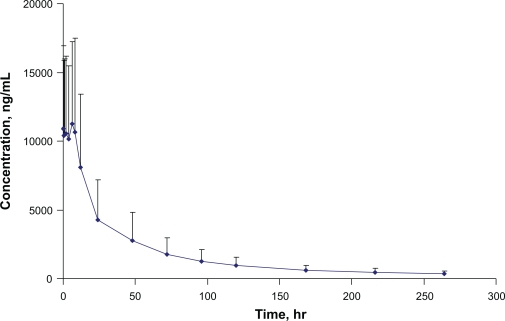
Plasma concentration—time profile of lumefantrine.

The validated method was used to study the pharmacokinetic interaction of LF and an antiretroviral in 13 healthy volunteers. After 6 doses of Coartem^®^ (480 mg LF twice daily), the plasma concentration—time profile over the period of 0–264 hr is shown in Figure 4. The precision and deviation for quality controls during analysis of LF samples is showed in Table 5. The method proved to be robust and reliable. The findings of this pharmacokinetic drug interaction study will be published separately.

## Conclusions

An HPLC-UV method for determination of LF in human plasma was developed based on a previously published method.^6^ Fine tuning of the sample preparation method improved the recovery of LF significantly. Gradient elution followed by a wash phase with a high percentage of organic solvent minimized interference from carry-over impurities. TFA (0.1%), instead of phosphate salt, added to the mobile phase minimized peak tailing. The internal standard halofantrine used is readily available from commercial sources. This method was validated based on the ACTG guidelines,^7^ which are based on FDA guidelines.

## Supplemental materials

Figure S1. Chromatograms of different sources of human plasma. Lumefantrine at LLOQ level and the I.S. were included as reference.

Figure S2. Chromatograms of potential concomitant drugs in the method. No significant peaks observed during the retention times for LF and I.S.

**Table S1.** Matrix effects from different sources of plasma (EDTA as the antocoagulant). Plasma samples spiked with lumefantrine at 900 ng/mL.

**Plasma (lot #)**	**Replicate**	**Anticoagulant**	**Conc. (LF) ng/mL**	**Theoretical conc (units) ng/mL**	**Mean ng/mL**	**SD**	**% CV**	**% Dev**
1 (23-08172)	1	EDTA	855	900	845	22.1	2.6%	−6.1
	2	EDTA	861	900				
	3	EDTA	820	900				
2 (23-08176)	1	EDTA	853	900	895	37.6	4.2%	−0.5
	2	EDTA	908	900				
	3	EDTA	925	900				
3 (23-08180)	1	EDTA	864	900	903	34.1	3.8%	0.3
	2	EDTA	918	900				
	3	EDTA	927	900				
4 (55-20429)	1	EDTA	950	900	947	17.2	1.8%	5.2
	2	EDTA	962	900				
	3	EDTA	928	900				
5 (23-06316)	1	EDTA	920	900	929	8.1	0.9%	3.2
	2	EDTA	930	900				
	3	EDTA	936	900				
6 (22-97035)	1	EDTA	899	900	939	36.5	3.9%	4.4
	2	EDTA	949	900				
	3	EDTA	970	900				

**Table S2.** Partial volumes precision and accuracy for lumefantrine.

**Nominal concentration: 30,000 ng/mL**			
	**Dilution**		
Sample #	4X	8X	12X
1	7126	3865	2399
2	7755	4134	2368
3	7663	4177	2290
Theoretical Conc.	7500	3750	2500
Mean	7515	4059	2352
SD	340	169	56
CV %	4.5	4.2	2.4
% Dev	0.2	8.2	−5.9
n	3	3	3

## Figures and Tables

**Table 1.** Recovery (RE) of lumefantrine and I.S. in the assay.

**Concentration (ng/mL)**	**Peak area (X10e4), n = 3**		**RE, %**

	**Clean sample (in MeCN-water)**	**Preextraction spiked**	
Low (120)	2.55 ± 0.04	2.36 ± 0.03	92.5
High (9000)	192.3 ± 2.2	161.3 ± 0.9	83.9
I.S. (25,000)	16.36 ± 0.29	13.83 ± 0.07	84.5

**Table 2.** Inter-day average back-calculated standard concentrations (n = 5).

**Theoretic Conc ng/mL**	**Mean ng/mL**	**SD**	**Precision (RSD, %)**	**Accuracy (% dev)**
50	53.6	4.3	8.1	7.2
100	104	4.3	4.1	4.4
250	252	16.2	6.5	0.6
500	511	14.8	2.9	2.1
1000	1020	15.7	1.5	2.0
2500	2504	44	1.8	0.2
5000	5013	82.8	1.7	0.3
7500	7629	105	1.4	1.7
10000	10146	105	1.0	1.5
Slope	0.0013	0		
Y-intercept	−0.0018	0.0064		
R	0.9998	0.0002	0.02	

**Table 3.** Intra-day and inter-day precision (% RSD) and accuracy (% dev) for analysis of lumefantrine in human plasma.

**Nominal, ng/mL**	**Intra-day**				**Inter-day**			

	**50**	**120**	**900**	**9000**	**20**	**120**	**900**	**9000**
Mean, ng/mL	48.9–57.5	123–131	890–957	9082–9555	54.1	126	921	9371
SD	0.83–2.55	2.30–8.37	10.6–32.6	221–449	3.4	5.3	36	343
RSD, %	1.4–5.2	1.8–6.7	1.1–3.7	2.3–4.9	6.3	4.2	3.9	3.7
% dev	−2.2–15	2.3–9.3	−1.1–6.4	0.9–6.2	8.2	5.1	2.3	4.1
n	5	5	5	5	25	25	25	25

**Table 4.** Stability of lumefantrine plasma samples and stocks.

**Storage conditions**	**LF level (ng/mL)**	**% Remaining**	**% RSD**
3 freeze/thaw cycles	Low (120)	99.5	3.0
	High (9000)	102.9	1.1
−70 °C for 9 months	Low (120)	99.4	3.8
	High (9000)	102.5	5.4
benchtop 6 days	Low (120)	100.6	2.6
	High (9000)	98.9	1.8
dry residue 18 hr	Low (120)	104.8	7.2
	High (9000)	93.9	6.0
dry residue 48 hr	Low (120)	57.4	8.1
	High (9000)	65.7	17
in LC solvent 48 hr	Low (120)	92.7	9.3
	High (9000)	95.3	4.3
−70 °C for 9 months	LF Stock solution	102	1.5
22 °C, 3 days	LF Stock solution	100	2.8

**Table 5.** Method performance during clinical sample analysis.

**QCs (ng/mL)**	**Low (120)** **n = 22**	**Medium (900)** **n = 22**	**High (9000)** **n = 23**	Extra-high* (24000) **n = 10**
Precision (% CV)	8.1	4.5	5.8	7.5
Accuracy (% Dev)	0.81	−0.03	4.3	−1.6

* Extra-high QCs were diluted by 3-fold.
