# Supplementary figures and images for: A Modified Method for Determination of Lumefantrine in Human Plasma by HPLC-UV and Combination of Protein Precipitation and Solid-Phase Extraction: Application to a Pharmacokinetic Study
Source: Anal Chem Insights. 2010 Mar 29;5:15–23. doi: 10.4137/aci.s4431 (PMC2865164; doi:10.4137/aci.s4431)

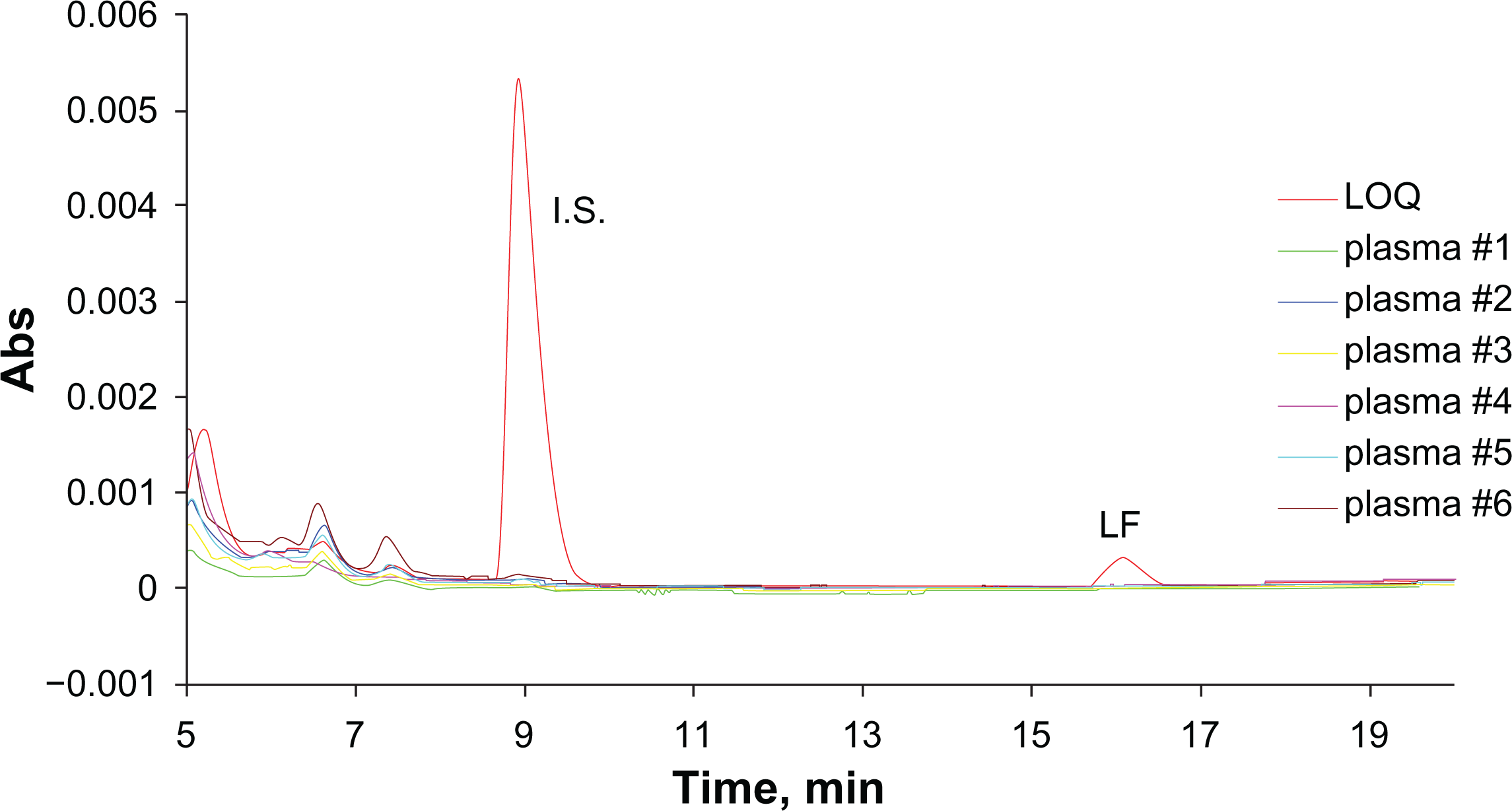

Supplement: Figure S1. — Chromatograms of different sources of human plasma. Lumefantrine at LLOQ level and the I.S. were included as reference. [file aci-2010-015f5.tif]

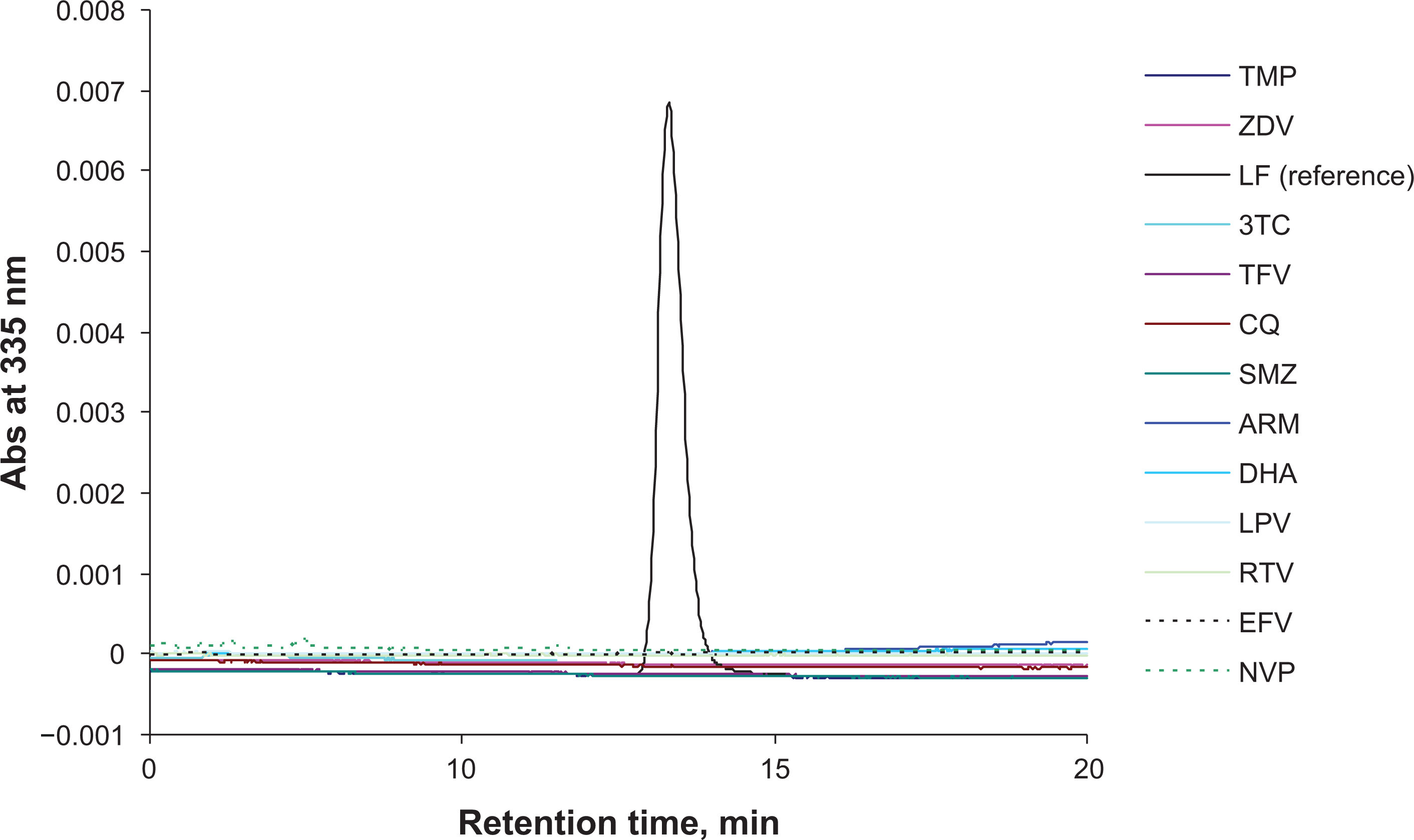

Supplement: Figure S2. — Chromatograms of potential concomitant drugs in the method. No significant peaks observed during the retention times for LF and I.S. [file aci-2010-015f6.tif]
